# Effect of Cilostazol on the Pharmacokinetics of Simvastatin in Healthy Subjects

**DOI:** 10.1155/2019/1365180

**Published:** 2019-01-09

**Authors:** Jung-Ryul Kim, Jin Ah Jung, Seokuee Kim, Wooseong Huh, Jong-Lyul Ghim, Jae-Gook Shin, Jae-Wook Ko

**Affiliations:** ^1^Department of Clinical Pharmacology and Therapeutics, Samsung Medical Center, Seoul, Republic of Korea; ^2^Department of Clinical Research Design & Evaluation, SAIHST, Sungkyunkwan University, Seoul, Republic of Korea; ^3^Department of Internal Medicine, Samsung Medical Center, Sungkyunkwan University School of Medicine, Seoul, Republic of Korea; ^4^Department of Clinical Pharmacology, Inje University Busan Paik Hospital, Busan, Republic of Korea

## Abstract

**Purpose:**

We evaluated potential drug-drug interactions between cilostazol and simvastatin, both CYP3A substrates, in healthy subjects.

**Methods:**

An open-label, two-period, fixed-sequence clinical study was conducted. Seventeen subjects were given a single oral dose of simvastatin 40 mg on day 1 and multiple oral doses of cilostazol 100 mg twice daily on days 2 to 5 followed by a single dose of cilostazol and simvastatin on day 6. Plasma concentrations of simvastatin and its active metabolite, simvastatin acid, were measured using liquid chromatography-tandem mass spectrometry for pharmacokinetic assessment. Moreover, serum lipid profiles under fasting conditions were determined.

**Results:**

The geometric mean ratios of the area under the plasma concentration-time curve from time zero to time infinity of simvastatin combined with cilostazol to that of simvastatin alone were 1.64 (90% CI, 1.38-1.95) for simvastatin and 1.31 (1.04-1.66) for simvastatin acid. In addition, coadministration with cilostazol significantly increased the maximum concentration of simvastatin and simvastatin acid, up to 1.8-fold and 1.6-fold, respectively. However, the effects of a single dose of simvastatin on serum lipid profiles were not affected notably when simvastatin was coadministered with cilostazol.

**Conclusions:**

Multiple doses of cilostazol increased the systemic exposure of simvastatin and simvastatin acid following a single dose of simvastatin.

## 1. Introduction

Cilostazol is a synthetic selective inhibitor of cyclic AMP phosphodiesterase III. It increases intracellular cyclic AMP levels and activates endothelial nitric oxide synthase [1], leading to a reversible inhibition of platelet aggregation and also antagonizing effects on vasoconstriction and cell proliferation [2]. Clinically, cilostazol is used for the treatment of patients with intermittent claudication and as myocardial protective agent in ischaemia-reperfusion injury [3]. Cilostazol is metabolized primarily by hepatic cytochrome P450 (CYP) 3A4 enzyme [4]. Furthermore, cilostazol is known as a weak clinical inhibitor of CYP3A and its inhibition constant for CYP3A4 was 6 uM from cDNA-expressed microsome study [5, 6].

Statins, known as 3-hydroxy-3-methyl-glutaryl-CoA reductase inhibitors, decrease endogenous cholesterol production by inhibiting the rate-limiting step in cholesterol synthesis [7] and thus are commonly used for the treatment of hypercholesterolemia [8]. Additionally, it was found that statin treatment was associated with superior leg function compared with no statin use, independently of cholesterol levels [9]. Claudication symptoms and walking performance were improved among patients treated with statins [10, 11]. Combination of atorvastatin with cilostazol had a synergistic effect on infarct size-limitation and on myocardial levels in rats through activation of endothelial nitric oxide synthase [12].

Simvastatin itself is an inactive prodrug and it is hydrolysed via esterases in plasma into the active *β*–hydroxyacid form, simvastatin acid [13]. Besides, it was found that CYP3A4 was the major CYP enzyme responsible for the metabolism of simvastatin to 3*∗*, 5*∗*-dihydrodiol, 3*∗*-hydroxy, and 6*∗*-exomethylene metabolites, which are inactive [13, 14]. Therefore, drugs that inhibit or induce CYP3A4 can significantly affect the plasma pharmacokinetic parameters of simvastatin. The CYP3A inhibitors, itraconazole and grapefruit juice, increased maximum concentrations (C_max_) of simvastatin by 12-fold and 17-fold, respectively [15, 16]. Moreover, it was reported that the majority of cases of rhabdomyolysis resulting from statin medications were associated with drug interactions [17].

To date, no studies have been reported regarding the pharmacokinetic interactions of cilostazol and simvastatin in humans, although the two drugs have been used concurrently in some patients. Both cilostazol and simvastatin are metabolized primarily through the CYP3A pathway, suggesting there may be potential drug-drug interactions. Therefore, we investigated the effect of cilostazol on the pharmacokinetics and lipid-lowering activities of simvastatin.

## 2. Methods

### 2.1. Subjects

Eligible subjects were healthy male volunteers aged between 20 and 49 years, with a body mass index from 19 to 27 kg/m^2^ at screening. The subjects were determined to be healthy by physical examination, vital signs, electrocardiograms, and clinical laboratory tests (haematology, blood chemistry, and urinalysis). Subjects were excluded if they had been exposed to any investigational drugs within 90 days prior to the first dose of study drug; if they used drugs known to induce or inhibit drug metabolizing enzymes; if they had known sensitivity to cilostazol and simvastatin; if they had an allergic disease requiring treatment; or if they had a positive result by serology for hepatitis or human immunodeficiency virus.

### 2.2. Clinical Study

**Figure 1:**
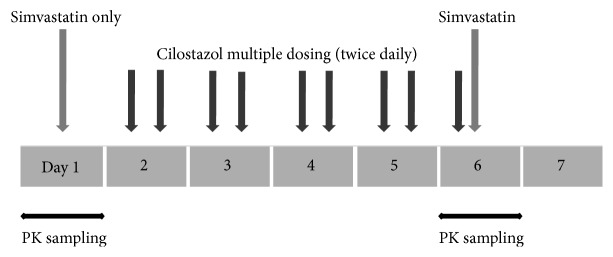
Illustration of the open-label, two-period, and fixed sequence clinical study with the administration of simvastatin (40 mg) and cilostazol (100 mg). PK is the abbreviation of pharmacokinetics.

This study was open-label, two-period, and fixed-sequence, where all subjects received two interventions in the same order (Figure 1). On day 1, a single dose of 40 mg of simvastatin was administered orally following an overnight fast of at least 10 hours. On days 2 through 5, oral 100 mg of cilostazol was administered twice a day. On day 6, at steady state in terms of cilostazol, a single dose of 40 mg of simvastatin was coadministered orally with the last dose of 100 mg of cilostazol after at least a 10-hour overnight fast. All simvastatin and cilostazol doses were administered with 240 mL of water. Apart from the water given with the drugs, subjects were not allowed to consume water within 2 hours after each drug administration. Subjects were confined to the Clinical Trial Center at Samsung Medical Center (Seoul, Republic of Korea), starting on the evening just before simvastatin dosing until the last 24 hour-blood sample was taken.

The study protocol was approved by the Institutional Review Board of Samsung Medical Center, and this study was conducted in accordance with the ethical principles of the Declaration of Helsinki (ClinicalTrials.gov identifier: NCT01383395) and Good Clinical Practice. All subjects provided written informed consent before enrolment.

### 2.3. Blood Sampling and Concentration Assay

Serial venous blood samples (8 mL) for pharmacokinetics were obtained through a 22-gauge indwelling catheter in a forearm vein before dosing and at 0.5, 1, 1.5, 2, 2.5, 3, 4, 5, 6, 8, 12, and 24 hours after simvastatin dosing in each period. Within 30 minutes after collection, samples were centrifuged at 1800 g for 10 minutes at 4°C. Separated plasma was collected in tubes and stored at –70°C until assay.

Plasma concentrations of simvastatin and simvastatin acid were measured using validated high-performance liquid chromatography with tandem mass spectrometry methods [18]. They were quantified over a theoretical concentration range of 0.2–50 *μ*g/L for both simvastatin and simvastatin acid. Assay interday precision and accuracy were 4.8-10.2% and 99.1-104.0%, respectively, for simvastatin, and 3.8-5.4% and 96.5-108.0%, respectively, for simvastatin acid.

### 2.4. Serum Lipid Measurements

To evaluate the lipid-lowering effects of simvastatin, serum total cholesterol, LDL-cholesterol, HDL-cholesterol, and triglyceride were measured before and 24 hours after simvastatin dosing in each period (on days 1, 2, 6, and 7) under fasting conditions. These lipid profiles were determined on a Hitachi 7600–110 chemistry analyser (Hitachi, Tokyo, Japan).

### 2.5. Safety Assessment

Adverse events (AEs) were recorded by means of spontaneous reporting by subjects and nonleading questioning throughout the study. Vital signs (blood pressure, pulse rate, and body temperature) were measured at regular intervals, and physical examinations were performed. AEs and any abnormalities in physical examination findings, vital signs, or clinical laboratory tests were assessed by investigators, who were not blinded to intervention. All AEs were coded according to the Medical Dictionary for Regulatory Activities (MedDRA® version 12.0).

### 2.6. Statistical Analysis

A sample size was calculated to ensure an adequate evaluation of the pharmacokinetic endpoint. Based on the literature on simvastatin [19], a sample size of 20 subjects could detect a 40% difference in systemic exposure between two interventions (simvastatin alone vs. simvastatin in combination with cilostazol) with a statistical power of 85% at a significance level of 0.05, allowing for a dropout rate of 20%.

The pharmacokinetic parameters for simvastatin and simvastatin acid were determined by a noncompartmental method using Phoenix® WinNonlin® 7 (Certara, Princeton, NJ, USA): area under the plasma concentration-time curve from time zero to time infinity (AUC_inf_); area under the plasma concentration-time curve from time zero to the time of the last quantifiable concentration (AUC_last_); C_max_; time to reach C_max_ (t_max_); and terminal elimination half-life (t_1/2_). Log-transformed AUC_inf_, AUC_last_, and C_max_ of simvastatin and simvastatin acid were compared between interventions using a mixed effects model, and the results were presented as the geometric least squares mean ratio (GMR) and 90% confidence interval (CI). Serum lipid profiles were compared using a mixed effects model. All statistical analyses were performed using SAS® Enterprise Guide® (version 7.1, SAS Institute, Cary, NC, USA), and statistical significance was defined at the 0.05 level by a two-sided test.

## 3. Results

### 3.1. Clinical Study

Twenty subjects were enrolled, and 17 completed the study. Three subjects withdrew their informed consent, one before any drug administration, one after the first simvastatin administration, and the other after the second day of cilostazol administration. Therefore, 19 subjects were included in the safety assessments, and 17 were included in the pharmacokinetics and lipid-lowering assessments. The subject characteristics are summarized in Table 1.

### 3.2. Pharmacokinetics

**Figure 2:**
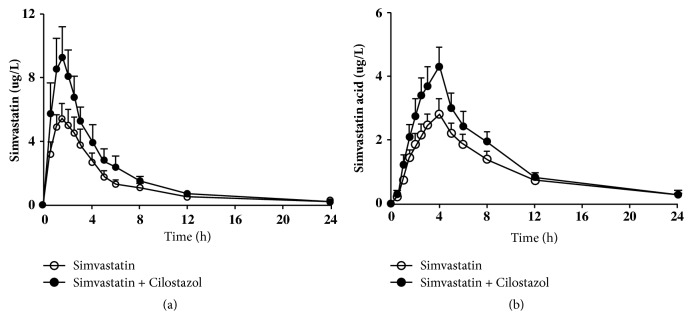
Mean plasma concentration-time profiles of simvastatin (a) and simvastatin acid (b) after single dose of simvastatin alone and with cilostazol in 17 healthy subjects. Bars represent standard error of measurements.

The mean plasma concentration-time profiles of simvastatin and simvastatin acid after administration of simvastatin alone and in combination with cilostazol are shown in Figure 2. There was considerable intersubject variability in plasma concentrations of both simvastatin and simvastatin acid.  Table 2 summarizes the pharmacokinetic parameters for simvastatin and simvastatin acid. The GMRs of AUC_inf_ of simvastatin coadministered with cilostazol to that of simvastatin alone were 1.64 (90% CI, 1.38-1.95) for simvastatin and 1.31 (1.04-1.66) for simvastatin acid. With the coadministration of cilostazol, the C_max_ of simvastatin and simvastatin acid increased 1.8-fold and 1.6-fold, respectively. However, the coadministration of cilostazol affected neither the t_max_ nor t_1/2_ of both simvastatin and simvastatin acid.

### 3.3. Serum Lipid Profiles

The serum lipid profiles under fasting conditions are shown in Table 3. There was no influence of cilostazol on the lipid-lowering properties of simvastatin. However, multiple doses of cilostazol reduced serum triglycerides significantly.

### 3.4. Safety

No serious AEs were reported, and no subjects discontinued the study due to AEs. The AEs are summarized in Table 4. All the AEs were considered related to the study drug. Most AEs were mild, except for moderate headache in two subjects. Headache was the most common AE, reported by 4 of 19 subjects receiving simvastatin and 12 of 18 receiving cilostazol. There were no clinically significant changes in vital signs or physical examinations.

## 4. Discussion

The present study was conducted to determine the effect of cilostazol on the systemic exposure to and lipid-lowering properties of simvastatin. Simvastatin and its active metabolite, simvastatin acid, are primarily metabolized by CYP3A. Therefore, simvastatin was selected as a substrate for assessing the potential for CYP3A inhibition by cilostazol and its metabolites.

In this study, cilostazol increased the C_max_ and AUC_inf_ of simvastatin by 1.8-fold and 1.6-fold, respectively. These were in line with the results of CYP3A substrate lovastatin coadministered with cilostazol, where the exposure of lovastatin increased 2-fold after multiple cilostazol doses [20]. However, the increment on the systemic exposure of simvastatin by cilostazol was relatively small compared with that of potent CYP3A inhibitors, itraconazole and grapefruit juice [15, 16]. In the present study, cilostazol did not affect the t_1/2_ of simvastatin, suggesting that the systemic exposure of simvastatin was altered by the reduced first-pass metabolism rather than the decrease in hepatic intrinsic clearance.

As for simvastatin acid, a 1.3-fold increase in AUC_inf_ was observed when simvastatin was coadministered with cilostazol. The previous study demonstrated that simvastatin acid was also metabolized primarily by CYP3A4/5 with a minor contribution from CYP2C8 in human liver microsomes, but simvastatin acid was a much poorer substrate than was simvastatin for CYP enzymes [21]. Similar to the t_1/2_ of simvastatin, that of simvastatin acid was not significantly different after coadministration of cilostazol with simvastatin. Therefore, smaller increases in the systemic exposure of simvastatin acid than in those of simvastatin might be attributed to the weak inhibition of CYP3A by cilostazol and alternative metabolic pathways.

A single administration of simvastatin produced no change in serum lipid profiles, and the increased C_max_ and AUC_inf_ of simvastatin by cilostazol did not affect lipid-lowering effects of simvastatin significantly. However, multiple doses of cilostazol reduced serum triglycerides significantly. Cilostazol was shown to improve dyslipidaemia in type 2 diabetic patients with peripheral vascular disease [22]. Another study in patients with intermittent claudication reported beneficial effects of cilostazol on serum lipid profiles [23]. The exact mechanism by which cilostazol reduces serum lipids is unknown. However, the effect of cilostazol on plasma lipoproteins might be due to inhibition of cyclic AMP [24]. Considering a single dose of simvastatin in healthy subjects, we cannot exclude the potential interactions of simvastatin and cilostazol. Multiple administrations of simvastatin with cilostazol may lead to the alteration in serum lipid profiles, requiring further investigation.

Headache was the most common AE in the present study, and this was compatible with the vasodilator action of cilostazol [25]. New-onset AEs were not reported following coadministration of simvastatin and cilostazol. Overall, the coadministration of simvastatin and cilostazol was well tolerated in healthy subjects.

There are several limitations to this study. Since pharmacokinetic interactions were assessed only in healthy subjects, these results may not be generalized to patients with dyslipidaemia. In addition, a single administration of simvastatin was not enough to determine the influence on lipid-lowering effects.

## 5. Conclusion

The systemic exposure of simvastatin and simvastatin acid was increased 1.6-fold and 1.3-fold, respectively, when cilostazol was coadministered. The clinical relevance of these pharmacokinetic interactions needs to be investigated in patients with the concurrent use of these drugs.

## Figures and Tables

**Table 1:** Subject demographics (n = 19).

Characteristic	Mean ± standard deviation	Median (min, max)
Age (years)	27 ± 6	25 (20, 41)
Body weight (kg)	67.6 ± 6.1	67.3 (55.6, 80.0)
Height (cm)	174.9 ± 6.3	177.0 (163.0, 182.4)
Body mass index (kg/m^2^)	22.1 ± 1.8	22.0 (19.0, 25.1)

**Table 2:** Pharmacokinetic parameters of simvastatin and its active metabolite, simvastatin acid, after a single oral administration of simvastatin 40 mg alone and in combination with multiple doses of cilostazol 100 mg in 17 healthy subjects.

Pharmacokinetic parameter	Simvastatin	Cilostazol + Simvastatin	Geometric least squares mean ratio	
			Point estimate	90% CI
*Simvastatin*				
C_max_ (*μ*g/L)	5.84 (69.7)	10.27 (62.0)	1.7581	1.4167, 2.1819
AUC_last_ (h·*μ*g/L)	19.97 (78.8)	34.06 (67.2)	1.7059	1.4456, 2.0131
AUC_inf_ (h·*μ*g/L)	23.15 (76.1)	38.02 (64.1)	1.6423	1.3812, 1.9527
t_max_ (h)	1.5 (0.5, 4.0)	1.5 (0.5, 4.0)	-	-
t_1/2_ (h)	3.73 (80.4)	3.74 (85.4)	-	-
*Simvastatin acid*				
C_max_ (*μ*g/L)	2.48 (63.4)	3.92 (58.6)	1.5840	1.3025, 1.9263
AUC_last_ (h·*μ*g/L)	17.81 (70.5)	25.62 (62.6)	1.4389	1.2127, 1.7072
AUC_inf_ (h·*μ*g/L)	23.08 (80.4)	30.27 (60.0)	1.3114	1.0363, 1.6596
t_max_ (h)	4.0 (1.5, 6.0)	4.0 (2.0, 6.0)	-	-
t_1/2_ (h)	5.97 (101.3)	5.39 (57.3)	-	-

Data are presented as geometric least squares mean (coefficient of variation), except t_max_ which is presented as median (min, max).

**Table 3:** Serum lipid profiles following a single dose of simvastatin alone on days 1 and 2 and in combination with cilostazol on days 6 and 7.

Variable	Simvastatin		Cilostazol + Simvastatin	
	Day 1	Day 2	Day 6	Day 7
	(n = 17)	(n = 17)	(n = 17)	(n = 17)
Total cholesterol (mg/dL)	164.5 ± 29.5	164.8 ± 29.7	159.0 ± 27.7	157.8 ± 28.8
p-value*∗*	-	1.0000	0.6053	0.5713
Triglyceride (mg/dL)	108.5 ± 41.8	99.6 ± 25.7	82.0 ± 19.6	78.2 ± 19.9
p-value	-	0.8124	0.0200	0.0126
LDL-cholesterol (mg/dL)	97.9 ± 27.9	99.4 ± 27.7	92.1 ± 25.9	92.8 ± 27.7
p-value	-	1.0000	0.4967	0.7611
HDL-cholesterol (mg/dL)	50.9 ± 12.5	49.6 ± 13.2	53.0 ± 12.0	53.5 ± 13.9
p-value	-	1.0000	0.7115	0.6426

Data are presented as arithmetic mean ± standard deviation.

*∗*p-value of comparison with Day 1 using a mixed effects model with the Bonferroni adjustment.

**Table 4:** Adverse events occurring with a single dose of simvastatin (40 mg) and multiple doses of cilostazol (100 mg) in healthy subjects.

Symptom and Sign	Simvastatin	Cilostazol	Cilostazol + Simvastatin
	(n =19)	(n=18)	(n=17)
Headache	4 (4)	12 (12)	-
Dizziness	-	1 (1)	-
Abdominal discomfort	-	1 (1)	-
Nausea	-	2 (2)	-

Data are presented as number of subjects experienced (number of events).

## Data Availability

The pharmacokinetic dataset analysed during the current study is available from the corresponding author on reasonable request.
